# Formation and Mechano-Chemical Properties of Chromium Fluorides Originated from the Deposition of Carbon-Chromium Nanocomposite Coatings in the Reactive Atmosphere (Ar + CF_4_) during Magnetron Sputtering

**DOI:** 10.3390/ma17205034

**Published:** 2024-10-15

**Authors:** Adam Roślak, Józef Doering, Wioletta Strzałka, Marcin Makówka, Anna Jędrzejczak, Łukasz Kołodziejczyk, Jacek Balcerzak, Łukasz Jóźwiak, Ireneusz Piwoński, Wojciech Pawlak

**Affiliations:** 1 Institute of Materials Science and Engineering, Faculty of Mechanical Engineering, Lodz University of Technology, 1/15 Stefanowskiego St., 90-537 Lodz, Poland; 223358@edu.p.lodz.pl (A.R.); marcin.makowka@p.lodz.pl (M.M.); anna.jedrzejczak@p.lodz.pl (A.J.);; 2 Department of Molecular Engineering, Faculty of Process and Environmental Engineering, Lodz University of Technology, 213 Wolczanska St., 93-005 Lodz, Poland; jacek.balcerzak@p.lodz.pl (J.B.);; 3 Department of Materials Technology and Chemistry, Faculty of Chemistry, University of Lodz, Pomorska 163, 90-236 Lodz, Poland

**Keywords:** fluorination of carbon nano-composite coatings, chromium fluorides, reactive magnetron sputtering in Ar + CF_4_ atmosphere

## Abstract

The literature analysis did not indicate any studies on fluorination tests of carbon nanocomposite coatings doped with transition metals in a form of nanocrystalline metal carbide in amorphous carbon matrix (nc-MeC/a-C). As a model coating to investigate the effect of fluorination in a tetrafluoromethane (CF_4_) atmosphere, a nanocomposite carbon coating doped with chromium-forming nanocrystals of chromium carbides in a-C matrix (nc-CrC/a-C) produced by magnetron sputtering from graphite targets and using a Pulse-DC type medium frequency power supply was chosen. After the deposition of the gradient chromium carbonitride (CrCN) adhesive sublayer, the fluorination of the main coating was conducted in a reactive mode in an (Ar + CF_4_) atmosphere at various CF_4_ content. It was observed that the presence of CF_4_ in the atmosphere resulted in a reduced amount of chromium carbides formed in favor of chromium fluorides. Thus far, this is an observation that seems unnoticed by the carbon coatings researchers. Fluorine was assumed to bond much more readily to carbon than to chromium, due to the stability of tetrafluoromethane (CF_4_). The opposite seems to be true. The mechanical properties (nano-hardness and Young’s modulus) and tribological properties in the ‘pin-on-disc’ friction pair are presented, along with the analysis of bonds occurring between chromium, carbon, and fluorine by means of X-ray photoelectron spectroscopy (XPS).

## 1. Introduction

Carbon coatings have been known for many years. Different bond contributions of sp2 or sp3 hybridization allow for various applications. In addition to polycrystalline diamond coatings (PCDs), there are typical amorphous a-C coatings and very hard tetrahedral ta-C coatings. All carbon coatings can be hydrogenated and the level of hydrogenation affects the tribological properties [1,2]. The addition of transition metals (W, Ti, Cr, and others) allows nanocomposite coatings to be obtained, where the carbides of these metals are nanometric in size and increase wear resistance in the friction contact [2,3,4,5,6]. The contribution of carbides, their type, and size enable control over the properties, e.g., mechanical [2]. Besides the typical carbide-forming metals, carbon coatings can be doped with non-metals, such as Si, O, N, or F [1,2,7]. While the effects of the first three mentioned elements are quite well described in the literature, there is little work on the influence of fluorine on carbon coatings. Donnett’s 1998 review paper [1] includes a summary of contemporary work on fluorinated carbon coatings. However, it describes only a few reports [8,9,10,11,12,13] about the effect of fluorine on reducing surface energy, increasing stresses in fluorinated carbon coatings, friction, and wear of such coatings, most often produced by the RF PA CVD method, in the presence of hydrogen. The most interesting thing, however, based on [13], the existence of unbound fluorine in the coating was described meaning that not all fluorine radicals were chemically connected to the carbon matrix. Favorable tribological features of carbon coatings with low fluorine content (up to 20%) were also shown. Unbound fluorine to the matrix increases stresses in the coatings and limits the ability to transfer loads in the friction contact.

The authors of [7] pointed out that fluorination can relate to the whole thickness of the coatings as well as exclusively fluorinating of the surface of the finished coating in hydrogen fluoride plasma. Modifying only the surface of the carbon coating for frictional contact work will not yield the expected results as the thin film will be quickly removed. Adding fluorine to the growing volume of the coating allows the coating to perform longer due to the disclosure of new, fluorinated portions of materials, as well as obtaining other interesting features, e.g., low dielectric constant (low-k) materials for electronic applications. The authors of [7] highlight the small number of articles available about fluorination of carbon coatings produced not only by PECVD but also by reactive sputtering or from a PTFE target. The former method, by the nature of the gaseous precursors used, contains hydrogen in the atmosphere. These works concern the C-H-F system and not only the fluorination of a-C coatings.

In Ref. [7], published in 2011, the authors wrote that research interest in F-DLC coatings is low. Reviewing the literature in 2024, we come to the same conclusion. These coatings are not widely known and used. Si-DLC coatings are more often used, e.g., [14,15,16]. Known applications of fluorinated carbon coatings cover three areas: medicine, mechanics, and optoelectronics. Due to their bactericidal, anti-corrosion, friction-reducing, and hemocompatibility properties, they are used in the medical industry as antibacterial and cytotoxic coatings for surgical instruments, orthodontic appliances, blood-contact devices, endoprostheses, and implants. They can be used to modify nanoporous dialysis membranes. In mechanics, the properties of polymer-like fluorine-carbon features are used to decrease friction and reduce surface energy. A well-known application is using F-DLC coatings as superhydrophobic coatings that prevent the icing of aircraft components. In optoelectronics, due to their interesting electrical properties, they are used to produce triboelectric nanogenerators (TENGs) and other MEMS devices using nanoimprint lithography. There have been attempts to use F-DLC coatings as electron field emission materials. There are reports of initial attempts to use such coatings to cover food packaging to reduce the diffusion of shielding gases. Attempts are being made to modify the properties of graphene in the form of a G-F-DLC coating.

Manufacturing technologies for fluorinated coatings include primarily PA CVD methods, but also plasma immersion ion implantation, magnetron sputtering, arc evaporation, laser ablation, and other methods. 

The literature analysis did not indicate any studies on fluorination tests of carbon nanocomposite coatings doped with transition metals of the nc-MeC/a-C type. As a model coating to investigate the effect of fluorination in a tetrafluoromethane (CF_4_) atmosphere, a nanocomposite carbon coating doped with chromium nc-CrC/a-C produced by magnetron sputtering from graphite targets and the use of a Pulse-DC type medium frequency power supply was chosen [6]. After the deposition of the gradient CrCN adhesive sublayer, fluorination of the main coating was conducted in a reactive mode in an (Ar + CF_4_) atmosphere at various CF_4_ contents. 

It was observed that the presence of CF_4_ in the atmosphere resulted in a reduced amount of chromium carbides formed in favor of chromium fluorides. Thus far, this is an observation that seems unnoticed by the carbon coatings researchers. Fluorine was assumed to bond much more readily to carbon than to chromium, due to the stability of tetrafluoromethane (CF_4_). The opposite seems to be true. The mechanical properties (nano-hardness and Young’s modulus) and tribological properties in the ‘pin-on-disc’ friction pair are presented, along with the analysis of bonds occurring between chromium, carbon, and fluorine (XPS).

## 2. Materials and Methods

**Figure 1:**
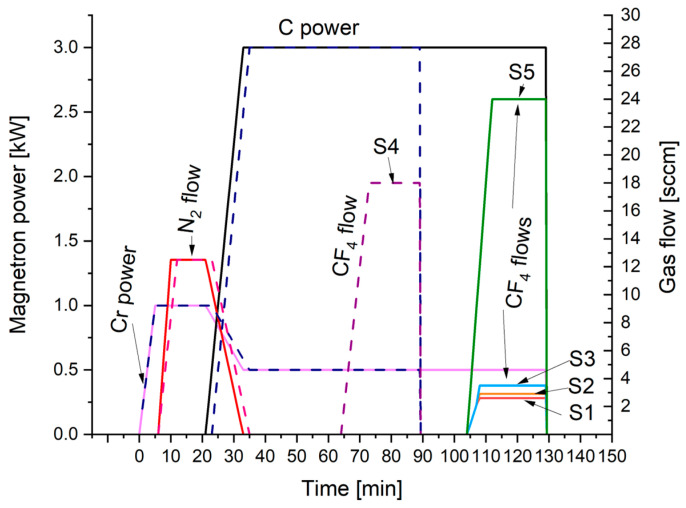
Deposition parameters (magnetron power and gas flow) with process time for fluorinated nanocomposite carbon coatings. The dotted lines refer to the shorter, S4 process.

Two types of samples were prepared. Vanadis 23 HSS steel samples (Uddeholm, Hagfors, Sweden), 26 mm in diameter and 6 mm in thickness were ground and polished for a mirror-finished surface. Polished (111) Si wafers were cut for 10 × 10 mm^2^ plates without other treatment. All samples were cleaned with detergent, for 10 min, with an ultrasonic bath of acetone, and then dried with clean, compressed air. A magnetron sputtering unit equipped with four balanced magnetrons with targets 107 mm in diameter and 10 mm in thickness was used. One target of pure chromium (99.95%) and three targets of sintered graphite (99.99%) were mounted onto magnetrons. For deposition, a substrate holder that allowed a one-fold rotation was used. After pumping to about 1.5 × 10^−3^ Pa, the ion cleaning in argon glow discharge was performed. Ion etching took 15 min under 3.7 Pa, 500 V of voltage, and up to 0.6 A of current. After 10 additional resting minutes, adhesive gradient interlayers, and main, fluorinated nanocomposite carbon coatings were deposited onto samples rotating at 4 rpm. The parameters of deposition are presented in Figure 1. The planned scheme for interlayer and the coatings was as follows: Cr–CrN–CrCN–nc-CrC/a:C–nc-CrC/a:C:F. The first step was a deposition of a pure chromium (Cr) adhesion improvement interlayer. Next, a CrN interlayer with a gradient increase of nitrogen content was deposited. After the deposition of the first two interlayers magnetrons with pure carbon targets were started, consistently increasing their power while reducing the amount of Cr sputtered and nitrogen introduced to the chamber. After half of the time of the main nc-CrC/a:C carbon-based nanocomposite coating deposition, tetrafluoromethane (CF_4_) was introduced into the deposition chamber with the volumetric flow rate ranging from 0 to 24 sccm. The total deposition process time was 130 min. One process was shorter, with a process time of 90 min. Five different processes were conducted varying the fluorine content in the outer zone of the coatings (designated as S1 to S5). One, additional process (designated as S0) was performed without CF_4_ addition, so it was just nc-CrC/a-C coating at the top of the gradient Cr–CrN–CrCN interlayer. 

The thickness was determined from the images of the fracture cross-section of coating deposited on the silicon wafer using a JEOL JSM-6610LV SEM microscope (JEOL, Akishima, Japan) Chemical composition was measured from the top of the samples with the EDS attachment Oxford Instruments X-MAX N 80 (Oxford Instruments, High Wycombe, UK). Surface scans were taken from an area of 750 µm^2^. Aztec 4.4 software was used for EDS chemical composition calculations. Due to the small thickness of the coating and the nature of the method, the EDS signal was also generated from the silicon substrate. It was removed from the calculations of chemical composition. The C/F ratio was determined, leaving only these two elements for calculations. The chemical compositions of the coatings are those averaged over the entire EDS volume tested (several µm). The XPS measurements cover only a shallow depth of up to several nanometers. This is where the observed differences in the contents of individual elements may come from. The EDS results were accepted as valid, bearing in mind the described methodological differences and the obtained structure of the coatings. The EDS line scan of chemical composition onto coating fracture cross-sections was made with an FEI NovaNano SEM 450 microscope (FEI, Hillsboro, OR, USA) equipped with an EDAX Octane Pro detector (Edax AMETEK, Pleasanton, CA, USA) and TEAM™ software (TEAM™ Edax Enhanced, ver. 4.6, 2019). Measurements were conducted in a line mode, enabling the collection of spectra for the efficient analysis of compositional gradients. The EDS measurements were performed with an accelerating voltage of 20 kV. 

The XPS analysis was carried out with a Kratos AXIS Ultra spectrometer (Kratos Analytical Ltd., Manchester, UK) using monochromatic Al Kα X-rays source of excitation energy equal to 1486.6 eV. Photoelectron spectra were collected from several analytic areas, each measuring 300 µm × 700 µm. The power of the anode was set at 150 W and the hemispherical electron energy analyzer was operated at a pass energy of 20 eV for all high-resolution measurements. Charge neutralization was not applied, and the spectra were not calibrated, since the samples’ surfaces were sufficiently conducting. The XPS results were recorded after 30 s of 3 keV argon ion etching to remove surface residues. Evaluation of XPS data was conducted using Kratos Vision 2 software. The background subtraction was performed with the Shirley algorithm and the symmetric Gaussian:Lorentzian = 70:30 function was applied to minor components fitting in F 1s, Cr 2p, and C 1s, whereas main fluorine, chromium, and carbon peaks were fitted with the use of an asymmetric function, since they are reported [17] as a tailed towards higher binding energy side, due to photoelectrons energy loss phenomenon.

Friction tests were performed using the CSM THT tribotester (CSM Instruments SA, Peseux, Switzerland) with ‘pin-on-disc’ method on coated Vanadis 23 HSS steel samples. The friction radius was 10 mm, linear velocity was 0.1 m/s, load force was 1 N, and the test distance was 500 m (7950 rotations). The diameter of the counterbody ball, made of AISI 52100 bearing steel, was 6.35 mm. Tests were performed under room temperature (about 20 °C and relative humidity of 30–50%). After the friction tests, the wear tracks were measured using contact profilometry (HommelTester T1000, Hommelwerke GmbH, VS-Schwenningen, Germany) and optical profilometry (S-neox, Sensofar Metrology, Terrassa, Spain). Optical profiler studies were conducted in confocal mode. The objective magnification applied during the measurements was ×20. For each sample, eight different areas of the wear track were measured. Registered data, with 850.08 × 709.32 µm^2^ dimensions, were treated using an academic license of OriginPro Version 2020 Software (OriginLab Corporation, Northampton, MA, USA). Integrated worn volumes in the friction paths were used to calculate the volumetric coefficients of wear from the known formula:

K_w_ = V·F^−1^·S^−1^, (1)

where: K_w_—coefficient of wear [mm^3^·N^−1^·m^−1^], V—volume of wear [mm^3^], F—load force [N], and S—test distance [m]. The average wear rate values were estimated for all coatings based on the separate results. One sample for each coating was investigated. 

Hardness (H) and elastic modulus (E) were measured using the nanoindentation technique on the Nano Indenter G200 system (KLA Corporation, Milpitas, CA, USA) onto silicon samples with coatings. As a reference, the uncoated Si substrate was analyzed as well. For nanoindentation, a diamond Berkovich tip (Micro Star Technologies, Huntsville, TX, USA) and the continuous stiffness measurement mode were used. The tip shape was calibrated by conducting experiments on a fused silica standard and data were analyzed using the approach presented in the work of Pharr et al. [18]. At least nine experiments were performed on each sample at a strain rate of 0.05 s^−1^, a harmonic displacement of 2 nm, and a frequency of 45 Hz, and the results were averaged.

## 3. Results

### 3.1. Thickness and Chemical Composition

Measured thicknesses and the chemical composition of deposited coatings are shown in Table 1. All samples had similar thicknesses from about 850 nm for S4 to 1070 nm for S5 samples. The rest of the processes resulted in thicknesses between the values shown. The chemical composition shows the increasing amount of fluorine in the coating. Changes in the partial pressure of CF_4_ are reproduced in the observed fluorine trends. Three groups of the coatings were deposited. S1, S2, and S3 are considered as low concentrations of fluoride. S4 and S5 have higher concentrations, and S0 has no fluorine registered. The oxygen content should be appraised as low, and typical for magnetron sputtered coatings. 

**Figure 2:**
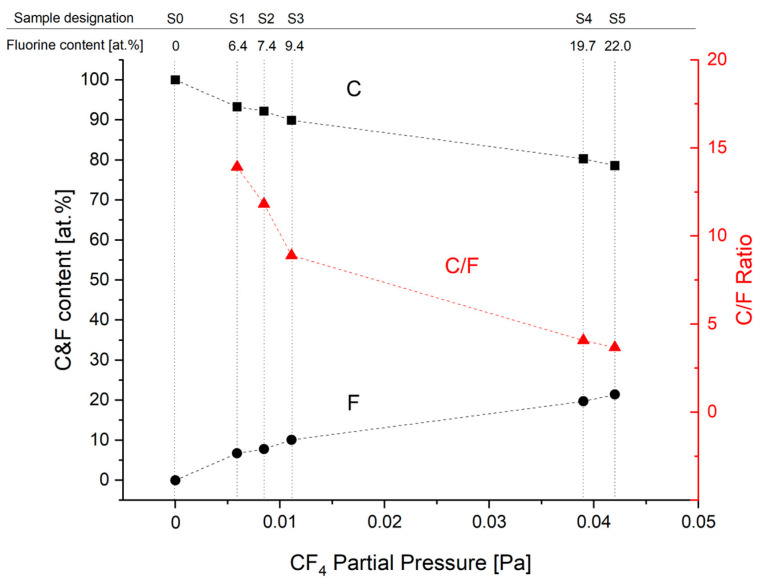
Carbon (■) and fluorine (●) concentration as well as C/F ratio (▲) of deposited coatings for different CF_4_ partial pressures. The lines are just guides for the eyes.

The changes in chemical composition influenced by different CF_4_ flows (represented as CF_4_ partial pressures) are shown in Figure 2, including the calculated C/F ratio. It shows differences in fluorine concentration for deposited coatings. 

**Figure 3:**
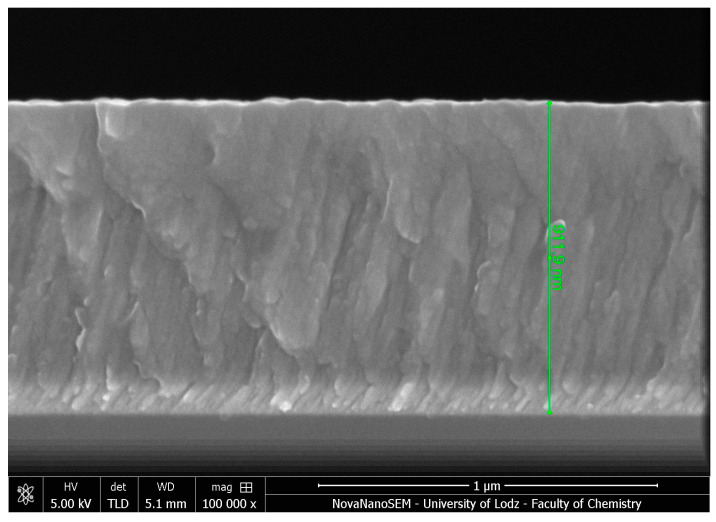
Fracture cross-sectional SEM image of S1 coating with 6.4 at.% of fluorine.

An example of a SEM cross-section view of an S1 sample is presented in Figure 3. It shows the dense microstructure of the coating along with a plain surface.

**Figure 4:**
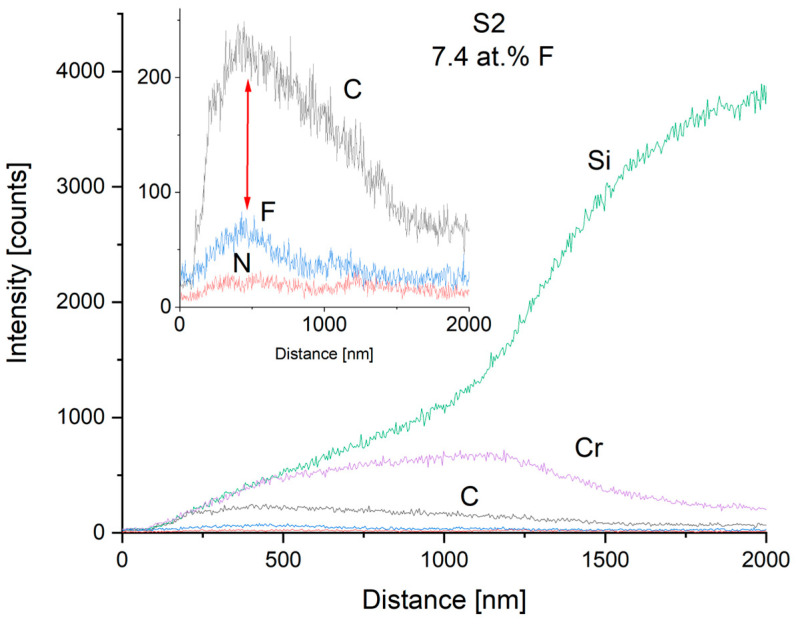
EDS line scan of the cross-section of S2 coating with low fluorine content (7.4 at.% F). The inset shows lines for light elements (C, F, and N).

**Figure 5:**
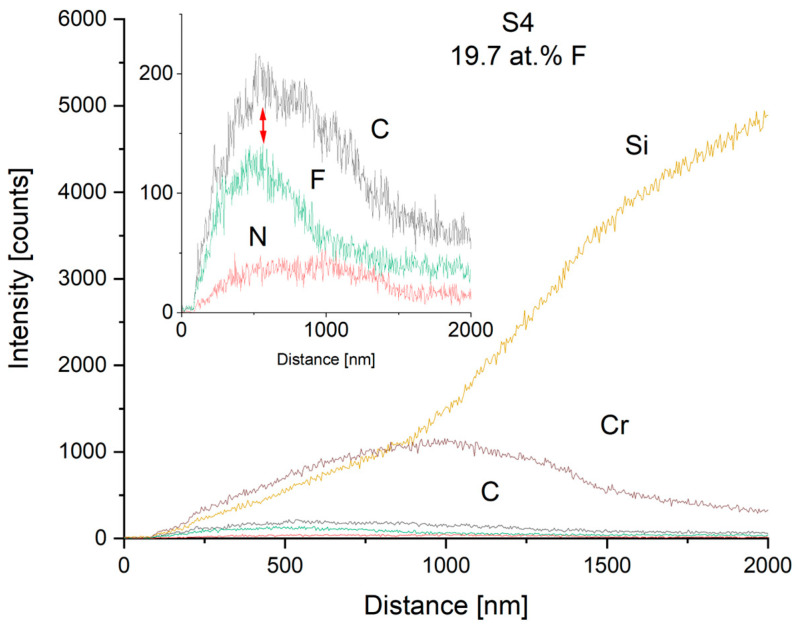
EDS line scan of the cross-section of S4 coating with high fluorine content (19.7 at.% F). The inset shows lines for light elements (C, F, and N).

To reveal the gradient Cr-CrN-CrCN interlayer and the fluorine depth of carbon nanocomposite coatings, SEM line scans onto fracture cross-sections were performed. The exemplary results for medium (S2—7.4 at.%) and high (S4—19.7 at.%) fluorine content samples are shown in Figure 4 and Figure 5, respectively. It is seen that the mutual ratio of carbon and fluorine content is in good agreement with the mean chemical composition measured from the plain surface. The depth of fluorine distribution corresponds to process parameters—it was planned to introduce CF_4_ at the outer part of the thickness of the carbon nanocomposite layer. The authors are aware that the EDS method is “rough” in terms of the electron beam spot size compared to the coating thickness, and the sensitivity for light elements, but qualitatively it turned out to be effective in exhibiting the differences between fluorine content in different samples (shown with arrows). The EDS method is known for low spatial resolution. The principles of the method are that the electron beam interacts with the sample and due to the high energy of electrons, the X-rays are generated from the substrate, from the depth of analysis of some micrometers. It means that the spot can be wider than the thickness of the coatings, usually about 2–3 µm. This is the reason why the signal spreads deeper than the actual coatings’ thickness. The signal from the Si substrate spreads also from the surface of the coating, so it was excited by the incident electron beam.

### 3.2. XPS

**Figure 6:**
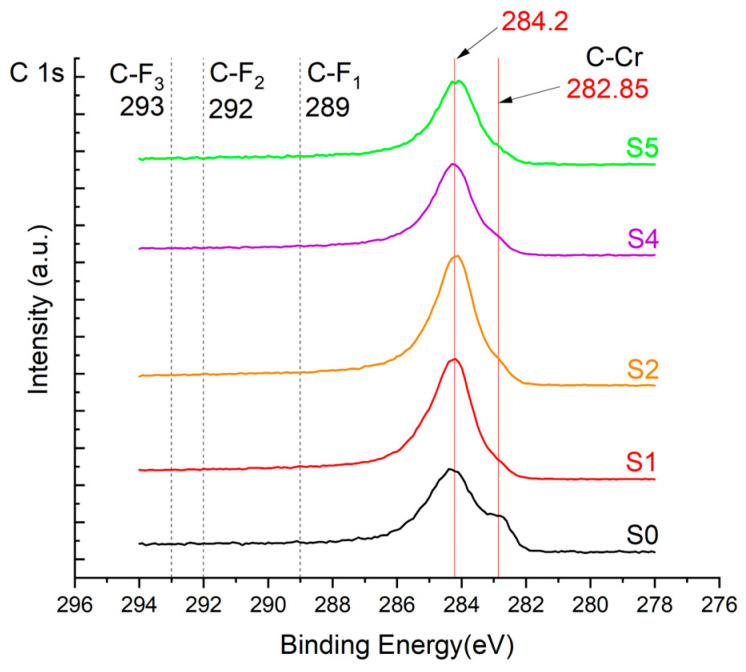
XPS C 1s spectra of the coatings with different fluorine content. Please observe the lack of C-F_x_ lines.

**Figure 7:**
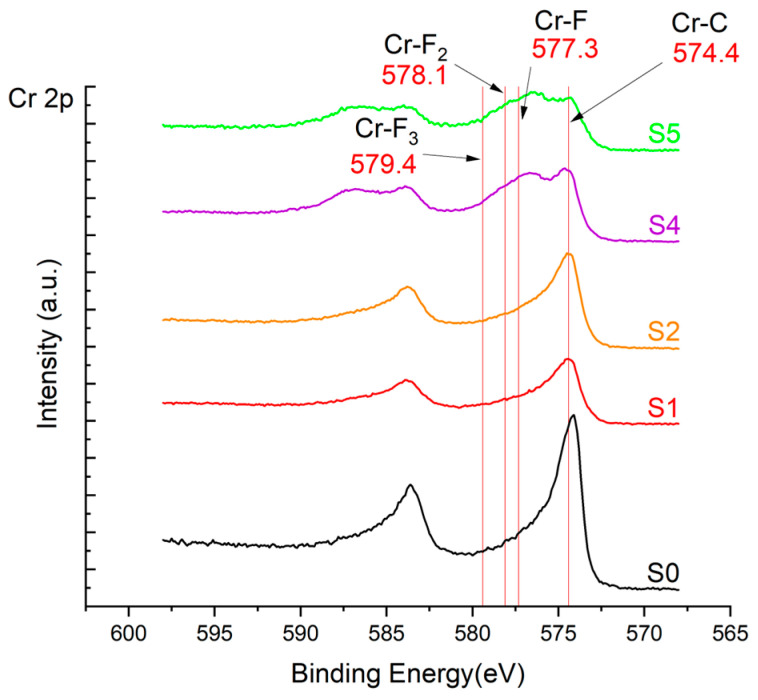
XPS Cr 2p spectra of the coatings with different fluorine content.

**Figure 8:**
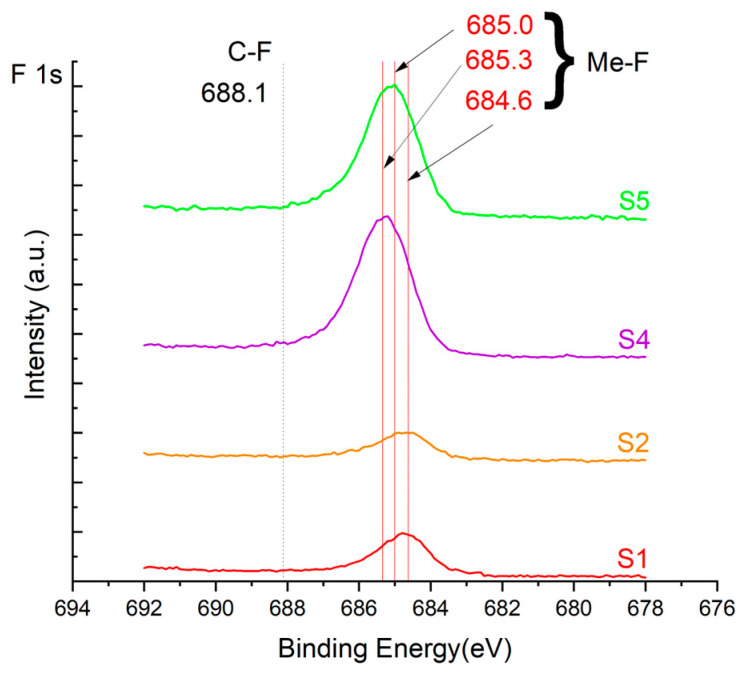
XPS F 1s spectra of the coatings with different fluorine content. Observe the lack of an F-C line.

The XPS results were analyzed in two ways. The first one is qualitative, shown in Figure 6, Figure 7 and Figure 8. In Figure 6, no C 1s peaks between carbon and fluorine were recorded at the expected positions of 289, 292, and 293 eV. A C-Cr peak at the known position of about 282 eV is seen for the S0 sample. For the fluoridized samples, its intensity diminishes to some extent. In Figure 7, one should notice an increase in the intensity of Cr-F_x_ bonds in the Cr 2p band with the increased fluorine content in the coatings. Figure 8 shows no expected C-F bonds on the F1s line at position 688.1 eV. 

The second way of showing data recorded with the XPS method was to fit individual component bands of the C 1s, Cr 2p, and F 1s lines. The results are presented in Table 2. The F 1s line was fitted with just one peak with the F-Cr band at positions close to 685 eV. The C 1s line of carbon is placed on standard and known energy of about 284.2 eV [19]. On the lower energy side of the carbon peak, at 282.85 eV, a well-described peak of C 1s from chromium carbide can be observed. Its intensity depends on the amount of chromium carbide in the material’s structure as shown, e.g., in [20,21,22,23,24,25,26]. Similarly, the Cr 2p spectrum for the S0 coating with 0% fluorine corresponds to the presence of chromium carbides [26]. Characteristic peaks representing bonds between carbon and fluoride were not detected for any of the specimens. No bonds were registered for C-F_1_, C-F_2_, and C-F_3_, where the energies have values of 289 eV, 292 eV, and 293 eV, respectively [27,28,29]. 

In the Cr 2p spectra (Figure 7 and Table 2), one can observe the vanishing of the main peak from the Cr-C bond at 574.4 eV in favor of peaks from Cr-F, which increase its intensity with growing fluorine content. According to [30], the Cr-F binding energies are 577.3 eV for Cr-F bonds, 578.1 eV for Cr-F_2_, and 579.4 eV for Cr-F_3_. A significant increase in the intensity of these peaks was observed for the investigated coatings S4 and S5 with a higher fluorine content (19.7 at.% and 22.0 at.% fluorine, respectively).

**Figure 9:**
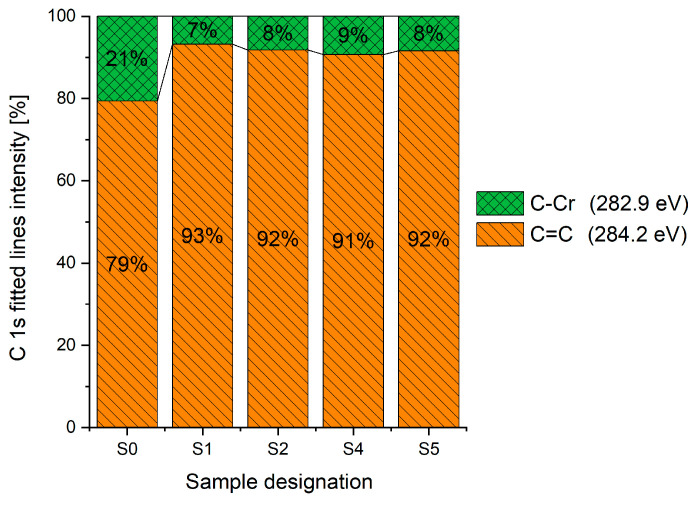
Carbon bond concentration based on XPS C 1s spectrum fitting.

**Figure 10:**
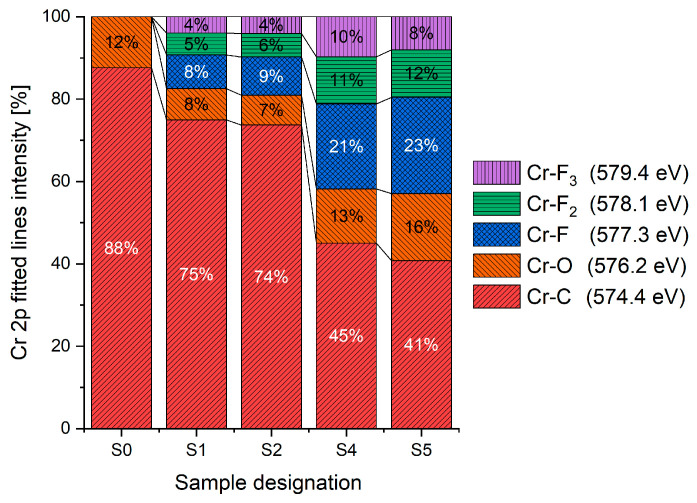
Chromium bonds concentration based on XPS Cr 2p spectrum fitting.

Analysis of binding energy spectra from the F 1s line is difficult, as very limited data was published with the registered shape of the F 1s line. Limited data are only available, for example, in Ref. [19]. Recorded spectra of F 1s for investigated coatings are presented in Figure 8 and Table 2. For higher amounts of fluorine in the coatings (S4 and S5), the F 1s peaks are transferred to higher energies of 685 eV and 685.3 eV, which is close to the peak at 684.6 eV for chromium fluoride. In general, many metal fluorides would have binding energies of about 684–685 eV. It should be mentioned that no clear peaks were registered in the range of C-F bonds at 688.1 eV. Such behavior can be explained by the preference of fluorine to bond with chromium instead of carbon atoms. The influence of fluorine action on C-Cr bonds is visible in Figure 9 and Figure 10, where the fitting components intensities are presented for C 1s and Cr 2p, respectively. High amounts of C-Cr bonds (21%) in the S0 sample were changed to 7–9% of such bonds in fluorided samples. In Figure 10, one can observe the increasing amounts of Cr-F_x_ bonds and diminishing Cr-C bonds. A clear band of 576 eV remains to be explained. It is standardly assigned to Cr-O bonds, but the low amount of oxygen in the coatings may suggest that there is a shift in the chromium bond at this position. It appears that the formation of chromium carbides is blocked by the presence of fluorine in the process atmosphere. Instead of with carbon atoms, the fluorine bonds with chromium and creates chromium fluorides of different stoichiometry. F 1s lines shifting to higher energies may be an indicator of this phenomenon. 

Binding energy spectra of carbon, chromium, and fluorine indicate combining fluorine with chromium instead of carbon. The expected bonding of fluorine with carbon was not registered, even though the fluorine came from a tetrafluoromethane compound, i.e., a combination of fluorine and carbon. It was discovered that fluorine combines more easily with chromium than with carbon supplied to the plasma from graphite targets and present in the atmosphere after CF_4_ dissociation. 

### 3.3. Friction and Wear

**Figure 11:**
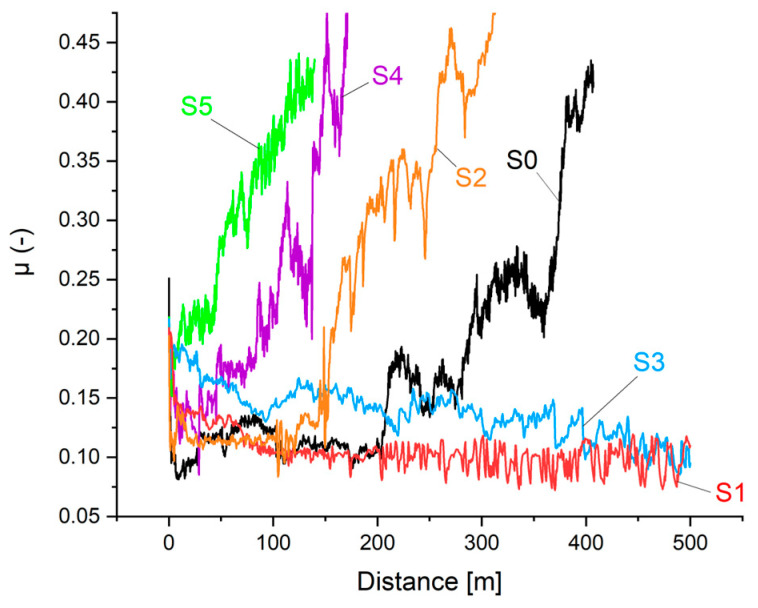
Friction coefficients for investigated carbon-based nanocomposite nc-CrC/a-C:F coatings with different amounts of fluorine.

**Figure 12:**
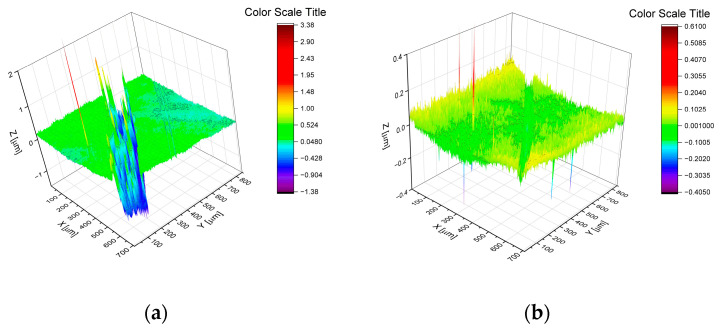
S0 (**a**) and S1 (**b**) profiles of wear path from optical profilometry. Note the Z-axis scale change.

The results of friction coefficients in the ‘pin-on-disc’ geometry are shown in Figure 11 and in Table 3. In Figure 11, one can distinguish three sets of curves. The shortest time of the coating’s life (when a coefficient of friction was lower than 0.15) was observed for two samples with the highest amount of fluorine (S4 and S5). The sample S0 (without fluorine) as well as the S2 sample (with 7.4 at.% of fluorine) were worn through after a medium time of friction (150–200 m). The best quality was observed for the samples S1 and S3 with 6.4 and 9.4 at.% of fluorine. A summary of frictional data (i.e., friction coefficient during the friction of the carbon layer, coating lifespan measured in meters, as well as calculated wear coefficients) is presented in Table 3. The value of the friction coefficient was lowest (0.10) for the coating S0. All investigated coatings with fluorine had a higher coefficient of friction probably because there are chromium fluorides built instead of fluorination of the carbon matrix. The highest coefficient of friction of 0.21 was registered for the sample S5 with the highest fluorine content (22.0 at.%). For the samples that pass the friction test (S1 and S3), the K_w_ values are appraised as a substantial value of wear for the coatings themselves. The order of magnitude of 10^−7^ mm^3^·N^−1^·m^−1^ is similar to other carbon-based nanocomposite coatings, for example in [6]. For worn-through samples (S0, S2, S4, and S5), the observed values of K_w_ show just mean values for destroyed coating systems. The exemplary views of the parts of friction paths from optical profilometry are shown in Figure 12 for samples S0 and S1, respectively.

### 3.4. Hardness and Elastic Modulus

**Figure 13:**
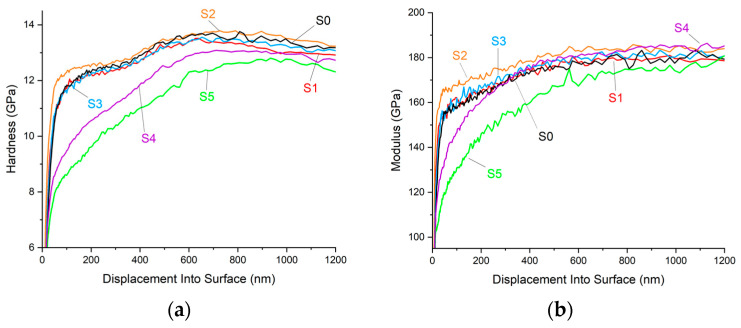
Hardness (**a**) and elastic modulus (**b**) curves from continuous stiffness mode of nanoindentation.

Hardness and elastic modulus are presented in Figure 13 and follow the behavior observed during friction. Two coatings with the highest fluorine content (S4 and S5) showed the lowest hardness and modulus. Sample S2, which was different from the expected friction vs. the chemical composition trend, was the hardest, even when the changes were quite small. It should be noted that the main carbon-based nanocomposite coatings were deposited onto a gradient Cr-CrN-CrCN adhesive interlayer. Observed gradients on hardness and elastic modulus curves in Figure 12 correspond to intentionally projected properties of such interlayer.

## 4. Discussion

The paper describes the influence of fluorination of known carbon-based nanocomposite coatings nc-CrC/a:C during reactive magnetron sputtering in an (Ar + CF_4_) atmosphere. The technological processes allowed for the coatings similar in thickness to be obtained. Chemical composition—mainly the fluorine content—followed the changes in the tetrafluoromethane flow during deposition. Surprisingly, chemical bonding analysis using the XPS method revealed no C-F bonds. Instead, spectra for the Cr-F bond with increasing intensity were registered. The expected formation of carbon fluorides was not present even though the fluorine introduced in the chamber originated from CF_4_—a compound with an already existing C-F bond. It was discovered that fluorine is more likely to bond with chromium than the carbon particles delivered from the graphite target or present in the atmosphere after the dissociation of CF_4_. The lowest coefficient of friction had S0—nc-CrC/a:C coating with no fluorine. This result showed that fluorination leading to a polymer-like carbon matrix with a lower coefficient of friction was not received. The addition of CF_4_ to the reactive atmosphere increased the Cr-F bond intensity. More chromium fluorides increased the observed coefficient of friction. The S2 coating with moderate amounts of fluorine (7.4 at.%) was different from the other, similar-in-composition (S1 or S3) coatings. Its hardness and elastic modulus were the highest. The coefficient of friction of the S2 sample (0.12) was close to the S0 coating (0.10). Its structure was stiffer and more brittle than other investigated coatings. A proper explanation of observed behavior needs more investigation. S1 and S3 coatings were similar and had a higher coefficient of friction (0.14 and 0.18, respectively) than S0 samples. Its low wear coefficients of the range 10^−7^ mm^3^·N^−1^·m^−1^ are comparable to known carbon-based nanocomposite coating, as in, for example [6]. Samples S4 and S5 (with a high fluorine content) showed deplorable tribological behavior. Their hardness and elastic modulus were much lower than the coatings with the fluorine content below 10 at.% (S1, S2, S3). It can be explained that a high amount of fluorine in the reactive gas during deposition allowed for a high concentration of chromium fluorides in the coatings. 

Obtained results show the possibilities of the new mechanisms of chromium fluoride formation during reactive magnetron sputtering in carbon–chromium plasmas in (Ar + CF_4_) atmosphere instead of fluorination of carbon matrix. Assuming that the observed effect of transition metal (chromium) affinity to fluorine radicals in mixed Cr-C-F plasma is bigger than carbon affinity to fluorine, it could be used (after a proper set of investigations) for gettering fluorine radicals from other sources of fluorine, as output gases in microelectronics manufacturing devices, leading to lowering air pollution, or utilization costs, etc.

## 5. Conclusions

The effect of CF_4_ on nanocomposite carbon-based nc-CrC/a-C thin films during reactive deposition by magnetron sputtering was described. XPS analysis revealed that fluorine does not combine with carbon in nanocomposite nc-CrC/a-C coatings and is instead bound to chromium at the expense of chromium carbides. The route of fluorine action with carbon and chromium influences the mechanical properties of the coatings, changing the carbide content. The chromium fluoride formation increased the observed coefficient of friction concerning the nc-CrC/a-C coatings. Wear resistance of fluorinated carbon-based nanocomposite coatings of the 10^−7^ m^3^·N^−1^·m^−1^ range was similar to the known values for base coatings [6]. The hardness and elastic modulus of the coatings showed almost no change for the amounts of fluorine up to 9.4 at.% and a drastic drop at the content of about 22 at.% of fluorine in the carbon-based nanocomposite coatings. 

## Figures and Tables

**Table 1:** Thickness and chemical composition of deposited nc-CrC/a-C:F coatings measured by EDS and XPS method.

Sample	Method	C	Cr	F	O	Sample Thickness [µm] ±0.1
S0	EDS	84.4	13.1	0.0	1.8	0.9
	XPS	68.4 ± 0.4	24.4 ± 0.2	0.0	7.1 ± 0.2	
S1	EDS	78.5	14.7	6.4	0.4	1.0
	XPS	81.52 ± 0.03	11.9 ± 0.3	3.8 ± 0.2	2.8 ± 0.1	
S2	EDS	74.3	17.9	7.4	0.4	0.9
	XPS	77.4 ± 0.2	14.96 ± 0.01	4.9 ± 0.2	2.73 ± 0.07	
S3	EDS	74.6	15.6	9.4	0.4	0.9
	XPS	n.a.	n.a.	n.a.	n.a.	
S4	EDS	60.5	18.9	19.7	0.9	0.9
	XPS	58.9 ± 0.9	20.3 ± 0.3	19.0 ± 0.6	1.77 ± 0.06	
S5	EDS	62.4	14.9	22.0	0.8	1.1
	XPS	61.4 ± 0.2	17.9 ± 0.3	19.0 ± 0.4	1.75 ± 0.03	

**Table 2:** Fitted XPS spectra of C 1s, Cr 2p, and F 1s lines.

Sample	at.% F	C 1s	Cr 2p	F 1s
S0	0	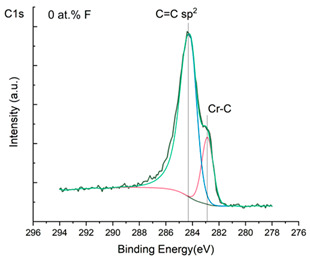	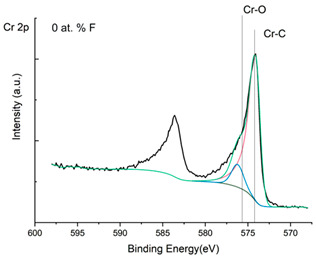	-
S1	6.4	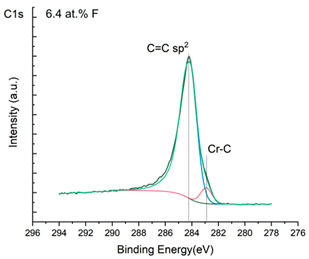	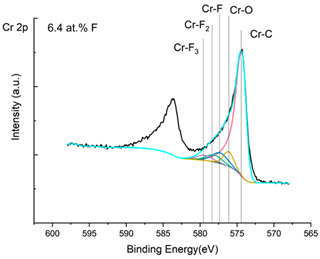	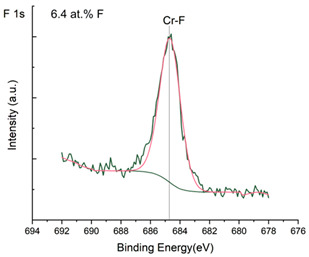
S2	7.4	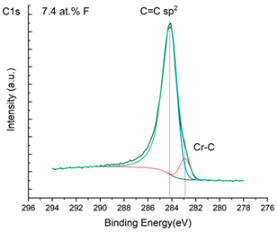	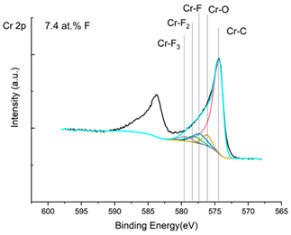	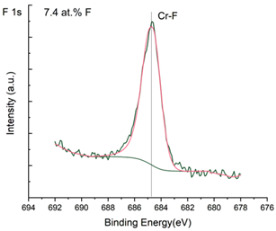
S4	19.7	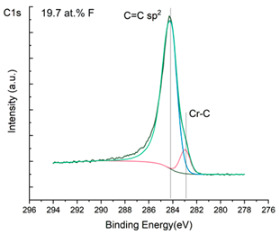	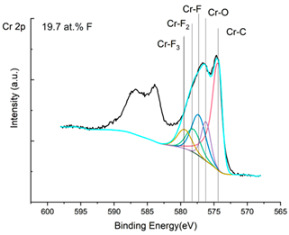	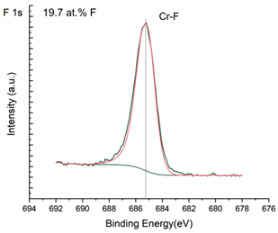
S5	22.0	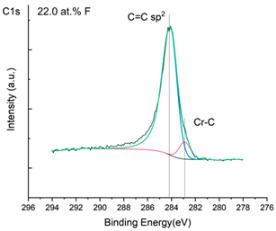	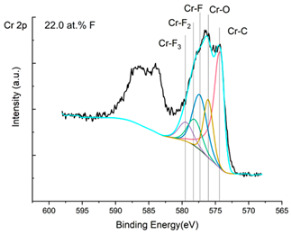	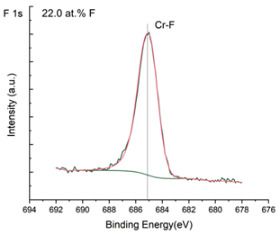

**Table 3:** Results obtained from the ‘pin-on-disc’ test.

Sample	Fluorine Content	Coefficient of Friction µ during the First 400 s (637 Rotations)	Lifespan of Carbon Coating	K_w_	
	[at.%]	[-]	[m]/[Rotations]	[mm^3^·N^−1^·m^−1^]	Std. Dev.
S0	0	0.10	202/3206	2.54 × 10^−5^	1 × 10^−5^
S1	6.4	0.14	500/7951	2.97 × 10^−7^	2 × 10^−7^
S2	7.4	0.12	143/2277	3.22 × 10^−5^	2 × 10^−5^
S3	9.4	0.18	500/7951	3.60 × 10^−7^	3 × 10^−7^
S4	19.7	0.14	45/714	7.84 × 10^−5^	2 × 10^−5^
S5	22.0	0.21	4/56	1.13 × 10^−4^	4 × 10^−4^

## Data Availability

The original contributions presented in the study are included in the article, further inquiries can be directed to the corresponding author.
